# Validation of the single-items Spanish-School Science Attitude Survey (S-SSAS) for elementary education

**DOI:** 10.1371/journal.pone.0209027

**Published:** 2019-01-02

**Authors:** Radu Bogdan Toma, Jesús Ángel Meneses Villagrá

**Affiliations:** Departamento de Didácticas Específicas, Universidad de Burgos, Burgos, Castilla y León, Spain; Universidad Nacional de Educacion a Distancia (UNED), SPAIN

## Abstract

The development of positive attitudes toward science is one of the main priorities in science education. However, there is a lack of reliable and valid instruments to measure Spanish-speaking elementary students’ attitudes towards school science. In this study, the translation and validation of the Spanish School-Science Attitude Survey (S-SSAS) is reported. The instrument was administered to 643 students enrolled in 3^rd^ to 6^th^ elementary grades. Psychometric evaluation of the S-SSAS provided sound evidence for validity (face, content, construct and criterion) and reliability (internal consistency and temporal stability). Content validity was confirmed through a panel of experts who reached great consensus in linking items to attitudinal constructs, with an ICC = .956. Think-aloud interviews confirmed that students have easily understood and correctly interpreted all items included, thus providing face validity for the S-SSAS. Consistent with theoretical expectations, predictive validity ranged between -.334 to 543 and concurrent validity was examined through S-SSAS correlation with two external measures of conceptual convergence that ranged from.301 to .560, thus confirming criterion validity. Construct validity was assessed by obtaining consistent results with the original scale in terms of reporting no statistically significant differences in attitudinal profiles towards school science between girls and boys and between students from urban and rural schools. Cronbach αfor the entire scale was .704, with item-total correlation ranging from .243 to .560, which reports acceptable internal consistency. Temporal stability with a 10-days span was good, with ICC = .873 and *r* = .464–790. Taken together, these results indicate that the Spanish single-items School-Science Attitude Survey is easy to administer and equally interpreted by both girls and boys enrolled in rural and urban elementary schools, thus being a valid and reliable instrument for measuring attitudes towards school science.

## Introduction

In the last decade, there have been significant changes in science education post-compulsory courses enrolments, with a steady decline in students interested in Science, Technology, Engineering and Mathematics (STEM) disciplines [1–3]. Therefore, the promotion of positive attitudes towards STEM disciplines is considered nowadays a priority objective in science education [4–7].

To date, attempts to measure attitudes have mainly focused on the administration of Likert-type scales that not always reported adequate psychometric properties and that often were too extensive to be implemented at elementary education stage. For example, the instruments with the strongest psychometric properties from Blalock et al. review [8] were validated with secondary school students [9–11] and posed administration problems due to their length [10]. Nonetheless, there are few newer instruments to measure attitudes toward science specifically developed and validated for elementary education: the 30-item BRAINS instruments [12], the 28-item Three-Dimension Elementary Science Attitude Survey [13], the 28-items attitude toward STEM instrument [14] or the 30-item Attitudes toward Science Class instrument [15]. However, some of these scales are focused on the measurement of attitudes towards science in general and are still to extensive, especially for longitudinal studies where educational interventions intended to improve attitudes are the main focus and therefore multiple data collection are needed [16].

This problem is further accentuated for scales in Spanish, with a great absence in the literature of validated scales for elementary stages. For example, some studies [17,18] have used extensive questionnaires in Spanish in both elementary and secondary schools, however, authors did not report on their psychometric properties. On the other hand, although Navarro, Förster, González, & González-Pose [19] have validated Fraser’s TOSRA scale [11,20] for Spanish-speaking students, the questionnaire is made up of a total of 70 Likert-type items, is focused on measuring attitudes towards science in general, and has also been validated only with students in higher grades than the Primary Education stage. Therefore, there is a need for valid and reliable quantitative instruments of easy and quick administration that facilitates the study of students’ attitudes toward school science at this stage. Consequently, the aim of this paper is to study the translation and validation of the Spanish single item instrument proposed by Kennedy, Quinn, & Taylor [21] called School Science Attitude Survey (SSAS). Considering the potential usefulness of brief measures of attitude toward school science, this study aims to provide sound evidence for the translation procedure used (face & content validity), for construct and criterion validity, and for internal consistency, sensitivity and temporal stability reliability.

## Theoretical underpinning of attitudes toward science

Although studies about students’ attitudes toward science have been mounting in the last decades, what is meant by attitudes toward science is still “(…) somewhat nebulous, often poorly articulated and not well understood” [6] p.1049, and no clear definition have been provided yet. Klopfer [22] offered insight about the concept under study by categorizing the attitude construct as a set of affective behaviors toward science as an enterprise, scientist, scientific inquiry, scientific careers and towards science-related activities in general. Gardner [23] provided further clarity by addressing the differences between «scientific attitudes», conceived as those elements inherent to scientific thinking and research, and «attitudes toward science», conceptualized as the sociological, psychological and affective conceptions and beliefs about science. Research developed at elementary level reported the existence of many sub-constructs underlying the attitudes toward science construct, including (i) students affective feelings and cognitive judgments of science [13], (ii) unfavorable outlook of science [24] and (iii) perception of scientists and value of science to society [25], among many others.

Recent results of past research indicating that, in general, students tend to have positive attitudes toward science but negative attitudes toward school science, stressed the need of advancing research about factors affecting students’ intentions and likehood of enrolling in science related activities and careers. Therefore, there is a shift from focusing on attitudes toward science in general, to focusing on attitudes toward school science, which may be a better predictor of student’s behavior than attitudes toward science in general [6]. Consequently, recent studies adopted various psychological theories like the Theory of Planned Behavior (TPB) [26] or the Expectancy-value theory (EVT) [27–31] to further explore student’s motivation, choice and persistence in studying science-related subjects and careers. Thus, attitudes are being studied by examining students enjoyment, self-efficacy, perceived difficulty [21] and others behavioral beliefs about the consequences of engaging in science [8,12,24,32].

## Literature review

### Existing attitude instruments for Spanish-speaking elementary students

Research on attitudes towards science has been dealing with methodological issues caused by a lack of rigor in the development and use of attitude measurement instruments. Munby [33]first concluded that authors do not take the necessary steps to develop valid and reliable attitude toward science instruments. Thus, for example, the presence of discrepant and contradictory results is very common in attitude toward science research and may be explained by the lack of psychometrically robust instruments [34]. In more recent years, Blalock et al. [8] underlined these issues by concluding that the vast majority of attitude instruments used in science education research the area of scientific education are lacking in terms of validity and reliability psychometric properties evidences.

Attitude toward science research conducted in Spain is no stranger to this problem. The most relevant attitude studies in Spain have used questionnaires that have not been subjected to reliability and validity tests and therefore, the extent to which the results reported in these studies are valid and reliable remains unclear (Table 1).

**Table 1:** Psychometric quality of the instruments used in Spanish attitude toward science studies.

*Instrument*	*Authors*	*Items and constructs*	*Grades*	*Reliability*	*Validity*	*Sensitivity*
PANA	De Pro Bueno & Pérez Manzano [17]	6 items measuring 6 constructs	6–10	No	No	No
	Pérez Manzano & De Pro Bueno[35]	17 items measuring 6 constructs	6–10	No	No	No
ROSE	Marbá-Tallada& Márquez Bargalló[36]	16 items measuring 3 constructs	6–10	No	No	No
	Pérez-Franco & De Pro Bueno[37]	19 items measuring 3 constructs	10	No	No	No
	Vázquez-Alonso &Manassero-Mas[38]	24 items measuring 3 constructs	4—undergraduates	No	No	No
	Vázquez-Alonso &Manassero-Mas[39]	80 items measuring 4 constructs	10	No	No	No
	Vázquez-Alonso &Manassero-Mas[40]	149 items measuring 5 constructs	10	No	No	No
	Vázquez-Alonso &Manassero-Mas[41]	16 items measuring 4 constructs	10	Yes	Yes^1^	No
	Vázquez-Alonso &Manassero-Mas[42]	24 items measuring 3 constructs	4–12	Yes	No	No
COCTS	Vázquez-Alonso, Acevedo Díaz, Manassero-Mas & Acevedo Romero[43]	202 items measuring 28 constructs	11–12	No	No	No
WAREING and PAC	Vázquez-Alonso &Manassero-Mas[44]	50 items measuring 10 constructs	8 -undergraduates	Yes	Yes^2^	No

^1^The Exploratory Factor Analysis revealed 3 items with cross loadings between factors.

^2^ Authors only provided evidence of content validity.

These studies have mainly used the ROSE instrument and the PANA questionnaire. In relation to the ROSE instrument, it is an adaptation of the Schreiner & Sjøberg [45]scale, originally developed in English and subsequently translated into several languages, including Spanish. The PANA questionnaire has been specifically designed by the authors. In both cases, the lack of validity, reliability and resistivity evidences is worryingly absent. None of the studies using the PANA questionnaire have provided information on their psychometric properties, and only one study of the six included in the Table 1 using the ROSE questionnaire provided information on the validity and reliability of the instrument, albeit with clear methodological problems. For example, Vázquez-Alonso & Manassero-Mas [41] have submitted the ROSE questionnaire to Exploratory Factor Analysis (EFA), obtaining cross loadings on three items. Instead of eliminating these items and redoing the factor analysis, the authors decided to keep the items, which is clearly a methodologically inadequate practice [46,47]. In short, these results demonstrate the need to develop psychometrically valid instruments, especially for the Spanish context.

### The school science attitude survey (SSAS)

The SSAS [21]is a web-based visual-analogue scale designed to examine student’s attitudinal profile (AP) to the area of school science through ten items that addresses the six common attitudinal constructs (AC) used in the literature of attitudes toward science (Table 2).From an initial pool of 46 items based on existing instruments and 22 newly developed, authors selected those that best represented each construct based on interviews with target sample, internal constancy results, and four dimensionality tests.

**Table 2:** The original SSAS [21].

*Attitudinal construct*	*Item*
(I) Intention to enroll in further science	1. I am very likely to enroll on a science course in Year 11(LT)
(E) Enjoyableness of school science	2. I think science is (SD boring–fun)
(D) Perceived difficulty of school science	3. I struggle with completing the assignments for science class (LT)
(S) Perception of self-efficacy in school science	4. I think I am very good at science (LT)
(U) Usefulness of science to careers $=\frac{us+up}{2}$	
(Us)Usefulness of school science to scientific careers	5. A job as a scientist would be interesting (LT)
(Up) Usefulness of school science to personal career choice	6. For my planned career, knowledge of school science will be (SD worthless–required)
(R) Relevance of school science $=\frac{rs+rp}{2}$	
(Rs) Relevance of school science to society	7. Science helps to make life better (LT)
(Rp) Personal relevance of School science $=\frac{Rp1+Rp2+Rp3}{3}$	
(Rp1) What do I want to learn about?	8. I want to learn about plants in my area (LT)
(Rp2)How applicable is school science to my everyday life?	9. For my everyday life, I think school science is (SD irrelevant–relevant)
(Rp3) Biological vs physical science	10. I want to learn about electricity and how it is used in the home

Four AC–intentions for future enrolment (I), enjoyableness (E), difficulty (D) and self-efficacy in school science(S)–were unidimensional and the remaining two AC–usefulness (U) and relevance of school science (R)–were found to be multidimensional.More specifically, usefulness of science for a future career in science (U_s_) and usefulness of science for personal career choice (U_p_) are the two dimensions underlying the usefulness (U) attitudinal construct. Finally, relevance to society (R_s_) and personal relevance (R_p_) of school science are the two dimensions underlying the relevance (R) attitudinal construct. Both the unidimensional and the dimensions underlying the multidimensional AC are measured through single-item measures with five response options, either Likert-type (totally disagree–totally agree) or semantic differential scales (i.e. fun–boring) with only extreme options being labeled.

The SSAS was selected for its translation and validation for Spanish speaking students for several reasons. Firstly, because it is consistent with recent recommendations on measuring attitudes toward the school science subjectinstead of science in general. In addition, it is an instrument that includes the main attitudinal constructs studied in this line of research. Finally, it is a short and easy to administer instrument, ideally for contexts in which time constraints limit the application of longer instruments, particularly in elementary education with younger students. Although SSAS uses single-items measures to examine all its constructs and sub-dimensions,which is in contrast to trends characterized by measuring a construct across multiple items, the initial validation results of the Kennedy et al. study [21] have shown that the SSAS reports robust results. Although multi-item instruments are more stable, reliable and accurate [48], single-item measures can be as psychometrically valid as long measures. For example, studies that have adapted extensive instruments into single-item scales have shown equally reliable and valid results as its multi-item version, such as the single item self-esteem scale [49]or the SIMP [50], which measures the Big Five personality with one item per construct.

## Method

### Psychometric properties

The quality of a measurement instrument is assessed through different psychometric tests that examines reliability and validity properties [51–54]. In the literature, different terminology and definitions are used to refer to the psychometric properties that should be examined during the scale validation process. The COSMIN initiative was an international effort in clarifying and standardizing the uses of the different terms related to psychometric properties of measurement instruments. Thus, through an international Delphi study, the COnsensus-based Standards for the selection of health Measurement INstruments (COSMIN) taxonomy was developed [55].

In this study we use the Polit-Yang taxonomy[56,57], which builds on the COSMIN study. In short, in the Polit-Yang taxonomy, reliability is defined as “(…) scores for people who have not changed are the same for repeated measurements, under several situations”[56] p. 25. The validity domain is defined as whether an instrument “(…) measures the construct(s) it purports to measure” [55] p.743.

**Fig 1:**
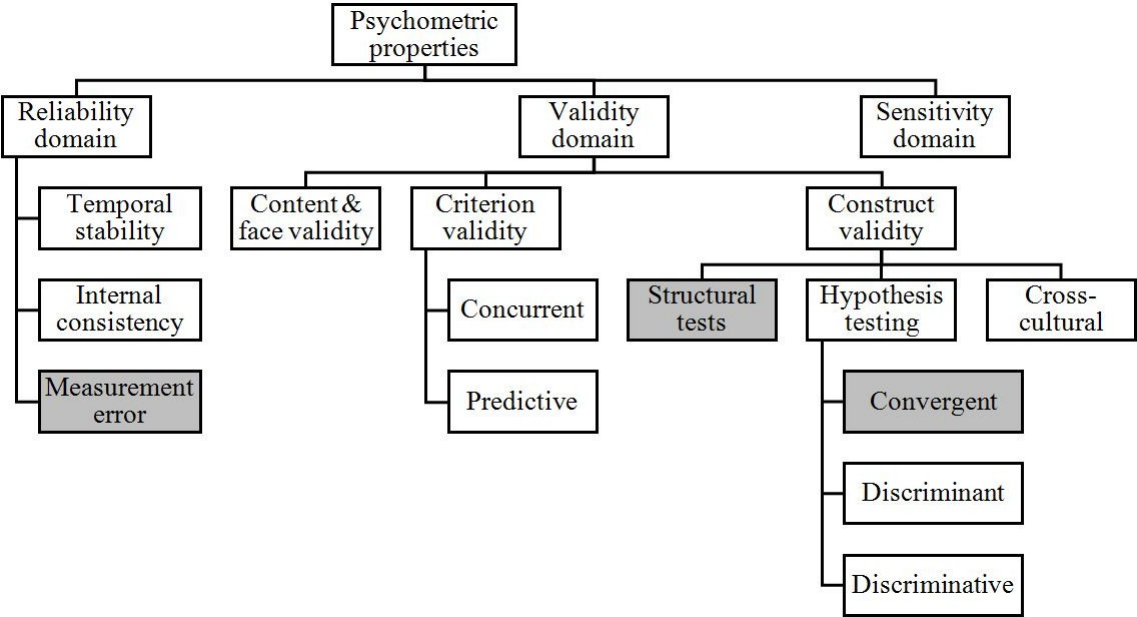
Theoretical framework for scale validation, based on Polit & Yang [56] taxonomy. Psychometric properties in grey quadrants were not measured in this study.

Relating reliability, the Polit-Yang taxonomy differs between (i) temporal reliability (i.e. weather scores are stable over time when traits have not changed), (ii), internal consistency (i.e. items measuring the same underlying construct), (iii) measurement error (i.e. error in score not related to true changes in the construct). As for validity, there are three components: (i) content and face validity, referring to “(…) the degree to which a content of an instrument adequately reflects the construct being measured” [57] p.1750, (ii) criterion validity (i.e. whether the proposed instrument is correlated with scores of existing instruments measuring the same constructs), and (iii) construct validity (i.e. if the construct under study is appropriately represented and conceptualized). Construct validity can be examined through structural validity tests like exploratory factor analysis (EFA) or confirmatory factor analysis (CFA), and through hypothesis testing validity tests, like convergent validity (i.e. items measuring the same constructs should be highly correlated), discriminant validity (i.e. constructs should measure different traits and therefore items from different constructs should be poorly correlated), and discriminative validity (i.e. whether the instrument discriminate between groups that are known to differ). For translated instruments, the cross-cultural validity should also be examined, which involves translation and back-translation and equivalent testing between the original and the translated version [58]. Finally, in addition to reliability and validity, an instrument should also provide evidence of sensitivity, meaning that it should be able to detect the spectrum of differences in the construct under study [59]. Fig 1 shows the theoretical framework adopted for scale translation and validation.

#### Sample and procedure

Participants were 643 students (47.9% girls, *M*_age_ = 10 years, *SD*_age_ = 1.26, range 8–13) enrolled in 3^rd^ (*n =* 75), 4^th^ (*n =* 142), 5^th^ (*n =* 174) and 6^th^ (*n =* 252) grades of 21 elementary education schools from Burgos, in Spain. Students were enrolled in both rural (48%) and urban (52%) schools. The Spanish version of the SSAS questionnaire was administered in a paper-pencil format upon students’ arrival at a week-long intensive curriculum enrichment program developed at an Innovative Education School Center located in Burgos.

Among all the participants, 117 students were randomly selected (52.1% girls, *M*_age_ = 9.32 years, *SD*_age_ = 1.14, range 8–13) for computing the concurrent validity of the scale and 88 students were randomly selected (46.6% girls, *M*_age_ = 9.55, *SD*_age_ = 1.41; range 8–12) for assessing the temporal stability of the Spanish SSAS instrument. No significant differences were found in neither age or in attitudes towards school science between the whole sample and both subsamples generated for concurrent and temporal stability validity.

Informed written consent was obtained from all participants’ parents.The study has been approved by the University of Burgos Education PhD Doctorate Commission and by the Vicerectorate for Research and Knowledge Transfer.

#### Measures

Two external measures were used to examine the criterion validity of the S-SSAS by exploring its relationship with two other attitude scales previously validated in the literature. The first instrument, named Scale of Attitudes Toward Science [60], measures three attitudinal constructs (i.e. positive affect toward science, self-confidence in learning science, students valuing science) through 7 Likert-type items. The second instrument, named «Arabic-Speaking Students’ Attitudes toward Science Survey» (ASSASS) [24] measures five attitudinal constructs (i.e. attitudes toward science and school science, unfavorable outlook of science, control beliefs about ability in science, behavioral beliefs about the consequences of engaging with science, and intentions to pursue science) through 32 Likert-type items. For this study, the «intentions to pursue science» subscale, composed of six items, was used. These two external measures were selected for computing criterion validity due to items and construct similarities with the S-SSAS (see S1 Table). For example, the «intention to pursue science» subscale of the ASSASS [24]is conceptually identical to the conceptualization of the “intention” construct from the S-SSAS. The «positive affect toward science» construct of the Sabah et al. scale [60] measures how much students enjoy science class (i.e. “I enjoy learning science” p. 695), sharing similarities with the enjoinment construct of the S-SSAS. The «self-confidence in learning science» construct of the Sabah et al. scale (i.e. “I usually do well in science, p. 695) is conceptually similar to the S-SSAS constructs «perceived difficulty of school science» and «perception of self-efficacy in school». Finally, the «students valuing science» construct of the Sabah et al. scale (i.e. I think learning science will help me in my daily life; I need science to learn other school subjects, p. 695) issimilar to the S-SSAS «usefulness of school science» and «relevance of school science».

## Results and discussion

### Translation and cross-cultural validity

The SSAS was translated into Spanish following a cross-cultural translation procedure [58]. In the first stage, a bilingual professor translated the SSAS from English into Spanish. In the second stage, another bilingual professor back-translated the Spanish version of the scale into the original language (i.e. English). In the third stage, both professors jointly reviewed the equivalence between the original and back-translated version suggesting minor modifications to the Spanish version: «Year 11» from the item assessing intention for future enrolment (i.e. I am very likely to enroll on a science course in Year 11) was translated as «E.S.O», which is the Spanish acronym that refer to the studies that begin at the end of Primary Education (i.e. middle school, students aged 12–16). In the fourth and final stage, six Elementary Education teachers were asked to verify that items wording were appropriated for the reading level of 3^rd^ to 6^th^elementary grade students. Consequently, the adjective «required» from the semantic differential item assessing usefulness of school science (i.e. For my planned career, knowledge of school science will be worthless/required)was translated as «useful», which is a better Spanish antonym for worthless. Original response format was maintained in all items, with the exception that the questionnaire was applied in a written rather than a web-based format. Original, Spanish and back-translation of the SSAS is outlined in Table 3.

**Table 3:** Original, translated and back-translated S-SSAS.

*Original SSAS*	*Spanish SSAS*	*Back-translation into English*
1. I am very likely to enroll on a science course in Year 11	Es muy probable que me apunte a Ciencias de la Naturalezaen la ESO	It is very likely that I will enroll in School Science course in ESO / Year 11.
2. I think science is (boring–fun)	Pienso que Ciencias de la Naturalezaes (aburrida–divertida)	I think school science is (boring–fun)
3. I struggle with completing the assignments for science class	Me cuesta terminar las tareas para la clase de Ciencias de la Naturaleza	I have difficulties in completing my homework for school science class.
4. I think I am very good at science	Pienso que soy muy bueno en Ciencias de la Naturaleza	I think I am very good at school science
5. A job as a scientist would be interesting	Un trabajo como científico sería interesante	A job as a scientist would be interesting.
6. For my planned career, knowledge of school science will be(worthless–required)	Para mis futuros estudios, el conocimiento de las clases de Ciencias de la Naturalezaes (inútil– útil)	For my future studies, knowledge of school science class will be (useless–useful).
7. Science helps to make life better	La ciencia ayuda a mejorar la vida	Science helps to improve life.
8. I want to learn about plants in my area	Quiero aprender sobre las plantas de mi entorno	I want to learn about plants in my surroundings.
9. For my everyday life, I think school science is (irrelevant–relevant)	Para mi vida diaria, creo que Ciencias de la Naturaleza es (poco importante–muy importante)	For my daily life, I think school science is (not important–very important)
10. I want to learn about electricity and how it is used in the home	Quiero aprender sobre la electricidad y saber cómo se usa en una casa	I want to learn about electricity and how it is used in a home.

### Content and face validity

Content and face validity was determined before large-scale administration using both a panel of experts and target population. An expert committee composed of a university professor in the field of psychology with extensive experience in instrument development and validation, and one university professor with expertise in science teaching and teacher training assessed the content validity of the translated version of the SSAS. Each expert was provided with the ten single-items scales comprising the SSAS and was asked to link each item to the attitudinal constructs or sub-constructs they consider to be measuring, and to evaluate how well each item represented the intended AC (i.e. 1-bad; 2-good; 3-great). Intraclass correlation (ICC) was used to examine agreement between experts in linking items to AC and for evaluating the representativeness of each item, which seems to be the most appropriate statistical method[61,62]. Following Koo & Li [63] guidelines for selecting and reporting Intraclass Correlation Coefficients, ICC estimates and their 95% confident intervals were calculated using the SPSS v.24 statistical software based on single rater, absolute agreement, two-way mixed-effects model.

Intraclass correlation coefficient (ICC) of .956 with 95% confident interval = .833 - .989 revealed a «good» to «excellent» inter-rater reliability in linking items to attitudinal constructs (AC). However, the psychology professor failed at assigning two items to the correct AC. More specifically, he assigned the item «I want to learn about plants in my area” to the «biological vs physical science» AC, and the item «I want to learn about electricity and how it is used in the home» to the «What do I want to learn about?» AC, instead of the other way around.

ICC coefficient of .800 with 95% confident interval = .408 - .945 revealed a «bad» to «excellent» agreement between the raters in evaluating each item representativeness (Koo and Li, 2016). While the professor with background in science teaching considered item «I want to learn about plants in my area» to be a great representativeness of the «What do I want to learn about?» AC, the psychology professor considered it to be only good. Taken together, these results seem to confirm that the SSAS has acceptable content validity.

After establishing the content validity of the S-SSAS, a cognitive interviewing approach known as Think-Aloud Protocol [64] was used with twelve students (three students from 3^rd^, 4^th^, 5^th^ and 6^th^ elementary grades, respectively) to assess face validity by examining student’s comprehensibility of the items and if their interpretation was similar to that intended by the researchers. Each student was given a copy of the survey and wasindividually prompted to explain what they think when reading and answering each item.

The Think-Aloud Protocol indicated that students have easily understood and correctly interpreted all items. However, some students found it difficult to understand two words. Thus, the adjectives «irrelevant–relevant» from the semantic differential item assessing personal relevance of school science (i.e. For my everyday life, I think school science is irrelevant/relevant) were worded in Spanish as «not important–very important», fostering understanding of lower grades students. No evidence of administration fatigue was identified, with most students taking approximately ten minutes (*M =* 9.16, *SD =* 1.05) to complete the task, time that is considerably reduced when students do not have to orally justify and explain their answers. A table with the researchers’ interpretation of the item and examples of students’ responses can be found in the S2 Table. Instructions for S-SSAS administration can be found in S1 File.

### Criterion validity

#### Predictive validity

Predictive validity was provided using Pearson product-moment correlation coefficient within S-SSAS attitudinal constructs. More specifically, consistent with the literature, the Spanish version of the SSAS would demonstrate criterion validity if there is a positive correlation between intentions and enjoyableness [65–67], between intentions and self-efficacy [68–70], and between usefulness and relevance [71,72].

As for predictive validity, the relationship within the expected S-SSAS AC reported medium to large Pearson’s *r* correlation coefficients that ranged from -334 to .543 (Table 4). More specifically, there was a strong-positive correlation between «intention to enroll in science courses» and «enjoyableness», *r* = .543, *p* < .01 and a medium-positive correlation between «intention to enroll in science courses» and «self-efficacy» *r* = .371,*p* < .01, «perceived usefulness» *r* = .357, *p* < .01 and «perceived relevance» *r* = .360, *p* < .01 attitudinal constructs. In addition, «enjoyableness of school science» was positively correlated with «self-efficacy» *r* = .414, *p* < .01, «perceived usefulness» *r* = .345, *p* < .01 and «perceived relevance» *r* = .311, *p* < .01 attitudinal constructs. As expected, «perceived difficulty» was negatively correlated with «self-efficacy» *r* = -.334, *p*< .01, and «perceived usefulness» was positively correlated with «perceived relevance» *r* = .431, *p* < .01. Taken together, these results are in line with theoretical expectations and previous literature results, thus confirming the criterion validity of the Spanish version of the SSAS.

**Table 4:** Pearson’s *r* correlation for criterion and construct validity and item-total correlation for internal consistency.

*Attitudinal constructs*	*Criterion and construct validity*										*Internal consistency*
	*1*	*2*	*3*	*4*	*5*	*6*	*7*	*8*	*9*	*10*	*Item-total r*
1. Intention	-	.543*	.171*	.371*	.357*	.360*	.552*	.338*	.225*	.298*	.560
2. Enjoyableness		-	.178*	.414*	.345*	.311*	.390*	.560*	.468*	.352*	.560
3. Difficulty			-	-.334*	.073	.053	-.107	-.236*	-.522*	-.037	.243
4. Self-efficacy				-	.224*	.232*	.215*	.169	.301*	.180	.501
5. Usefulness					-	.431*	.592*	.380*	.079	.428*	.410
6. Relevance						-	.416*	.356*	.160	.406*	.401
7. ASSASS							-	.397*	.076	.439*	
8. PATS								-	.264*	.268*	
9. SCS									-	.189*	
10. SVS										-	

*. Correlation is significant at the 0.01 level (2-tailed)

ASSASS refers to the intention subscale of Abd-El-Khalick et al. instrument[24]

PATS refers to positive affect toward science subscale of Sabah et al. instrument[60]

SCS refers to pelf-confidence in learning science subscale of Sabah et al. instrument[60]

SVS refers to students’ valuing science subscale of Sabah et al. instrument[60]

#### Concurrent validity

The relationship between S-SSAS’s «intention to enroll in further science»ACand ASSASS’s «intention to study science» subscale was measured. The concurrent validity of the remaining S-SSAS’s AC’s was assessed by studying the relationship between «enjoyableness of school science» S-SSAS’s AC and Sabah et al.’s «positive affect toward science» subscale; between «perceived difficulty of school science» and «perception of self-efficacy in school» S-SSAS’s AC and Sabah et al.’s «self-confidence in learning science» subscale; and finally, between «usefulness of school science» and «relevance of school science» S-SSAS’s AC and Sabah et al.’s «students’ valuing science» subscale.

In relation to concurrent validity, the relationship between S-SSAS AC’s and the scales developed by Abd-El-Khalick et al. [24] and Sabah et al. [60] reported medium to large Pearson’s *r* correlation coefficients that ranged from .301 to .560 for the expected attitudinal constructs (Table 4).

More specifically, there was a strong, positive correlation between «intention to enroll in science courses» AC’s of both scales, *r* = .552, *p* < .01, and between «enjoyableness» AC’s of both scales, *r* = .560,*p* < .01. Also, there was a strong, negative correlation between the S-SSAS «difficulty» AC and Sabah et al. [60] «self-confidence in learning science» subscale, *r* = -.522, *p* < .01. Finally, there was a medium, positive correlation between the «self-efficacy» AC’s of both scales *r* = .301, *p* < .01, «usefulness» AC’s of both scales *r* = .428, *p* < .01, and «relevance» AC’s of both scales *r* = .406, *p* < .01. These results seem to indicate that the S-SSAS shares satisfactory variance with other validated instruments intended to measure students’ attitudes toward science, thus further confirming the criterion validity of the S-SSAS.

### Construct validity

#### Discriminant validity

Discriminant and discriminative tests were used to provide evidence for hypothesis testing construct validity. Discriminant validity was examined using Pearson Product-Moment correlation coefficient within S-SSAS attitudinal constructs. Consistent with recent recommendations, moderately-strong correlation between factors are acceptable[73], however, correlation must be below the .80 cut off to consider that the translated S-SSAS provides evidence for discriminant validity [74].

Relating the discriminant validity of the S-SSAS, Pearson’s *r* correlation coefficient within S-SSAS attitudinal constructs were lower than the .80 cutoff (Table 4), thus discriminant validity is confirmed.

#### Discriminative validity

For discriminative validity, SSAS results were analyzed in terms of gender (girls and boys) and school type (rural and urban schools) differences to determine if they are consistent with the original SSAS results [21]. Specifically, independent sample *t* test with Bonferroni correction were computed using the SPSS v.24 statistical software[75].

The independent sample *t-*test revealed no statistically significant differences in attitudinal profiles towards school science between girls and boys (Table 5), neither between students from rural and urban schools (Table 6).

**Table 5:** Attitudinal profiles towards school science according to gender variable *SSAS*.

*SSAS constructs*	*Gender*	*M*	*SD*	*t-test*		
				*t-value*	*df*	*p*
Intention	Girls	3.17	1.09	1.41	436	.159
	Boys	3.02	1.15			
Enjoyableness	Girls	3.58	1.15	.58	436	.561
	Boys	3.52	1.18			
Difficulty	Girls	3.77	1.18	127	436	,204
	Boys	3.62	1.29			
Self-efficacy	Girls	3.48	1.05	- .845	436	,399
	Boys	3.57	1.03			
Usefulness	Girls	4.01	.81	.508	436	,612
	Boys	3.97	.93			
Relevance	Girls	3.76	.75	- 1.610	436	,108
	Boys	3.88	.76			

**Table 6:** Attitudinal profiles towards school science according to school type variable.

*SSAS constructs*	*Gender*	*M*	*SD*	*t-test*		
				*t-value*	*df*	*p*
Intention	Urban	3.08	1.10	-.397	436	.692
	Rural	3.11	1.16			
Enjoyableness	Urban	3.54	1.14	-.146	436	.884
	Rural	3.56	1.21			
Difficulty	Urban	3.71	1.25	.384	436	.701
	Rural	3.66	1.19			
Self-efficacy	Urban	3.46	1.03	-1.584	436	.114
	Rural	3.62	1.05			
Usefulness	Urban	3.97	.87	-.494	436	.622
	Rural	4.01	.88			
Relevance	Urban	3.83	.73	.209	436	.835
	Rural	3.81	.79			

Taken together, these results are consistent with the original SSAS [21], supporting that the Spanish SSAS items are understood and interpreted like-ways by both boys and girls, regardless of whether they are enrolled in rural and urban schools. This further corroborates the valid applicability of the Spanish SSAS.

### Reliability

#### Internal consistency

Cronbach’s α cannot be computed for single-item measures [53], and for multidimensional instruments, it should be computed for every construct [76, 77]. However, since the SSAS scale was originally developed to measures students’ attitudinal profile toward the school science construct, Cronbach’s α was computed for the entire scale, which should report tentative results about the internal consistency of the instrument. The α > .70 threshold for acceptable reliability was used [54]. To further examine internal consistency and provide more robust estimates than the Cronbach α, corrected item-total correlation for each of the six S-SSAS AC were calculated, with correlation above .3 suggesting good internal consistency [54,76, 78].

The Spanish SSAS reported good internal consistency (Table 4), with all corrected item-total correlations but the one measuring perceived difficulty of school science being far above the lower limit of .3 that has been suggested in the literature. Cronbach alpha for the entire scale was moderate (α = .704). This value can be considered acceptable for preliminary research. This results indicates that items included in this instrument does correlate very well with the overal scale assesing attitudes toward school science and therefore confirms that the translated SSAS has aceptable internal consistency.

#### Temporal stability

To assess the temporal stability of the Spanish version of the SSAS, a subgroup of students (*n* = 88) ranging from third to sixth grade of elementary education completed the scale at two times with a 10-days interval. A 10-days’ time span was used as it’s been considered in previous literature as adequate for computing test-retest reliability [79]. The first and second administration scale was done 10 days before and upon student’s arrival at the week-long intensive curriculum enrichment program, respectively. Pearson product-moment correlation coefficient was used to compute students’ responses between time 1 and time 2. In addition, as Intraclass correlation (ICC) seems to be more suitable than Pearson correlation coefficient when performing temporal stability reliability analysis [80], ICC was used to further estimate test-retest reliability by taking into account the measurement error [61]. More specifically, ICC estimates and their 95% confident intervals were calculated using the SPSS v.24 statistical software based on single ratter, absolute agreement, two-way mixed-effects model [63].

The relationship between each student S-SSAS global result at Time 1 and Time 2 revealed a significant large Pearson’s *r* correlation coefficients, *r* = .779, *p* < .01. According to each AC, the test-retest reliability of the S-SSAS ranged from medium to large significant Pearson’s *r* correlation coefficients (*r* = .464 - .790). In Table 6 Pearson’s correlations results between SSAS at Time 1 and Time 2 are reported. Examination of this table reveals that «enjoyableness of school science» is the AC with lowest test-retest reliability, and that «relevance of school science to society», and «intention to enroll in further science» AC’s reported the best temporal stability reliability.

ICC of .873 with 95% confident interval = .805 - .917 revealed a «good» to «excellent» test-retest reliability for the S-SSAS global results, with a mean difference between the two administrations (Time 2 –Time 1) of only .07. Intraclass coefficient results for each S-SSAS attitudinal construct confirms the temporal stability of the S-SSAS scale, with all but one AC (i.e. enjoyableness of school science) revealing a Intraclass correlation above .7 (Table 7).Among the S-SSAS constructs, «Enjoyableness of school science» has reported less test-retest reliability, suggesting that it may be an attitudinal construct that is more sensitive to classroom variables (e.g. science content, teaching methodology, classroom activities). In contrast, «relevance of school science to society» and «intention to enroll in further science» AC’s seems as the most stable over time, coinciding with earlier studies that show how difficult it is to change student’s intentions to enroll in a scientific career [81]. Overall, these results indicate that the Spanish version of the SSAS has a good temporal stability with a 10-days span between the first and second administration.

**Table 7:** Intraclass correlation between S-SSAS constructs at Time 1 and Time 2.

*SSAS AC*	*r*	*Intraclass* *correlation*	*95% Confidence Interval*		*Mean difference* *(Time 2 –Time 1)*
			*Lower Bound*	*Upper Bound*	
Intention	.771	.868	.798	.913	.11
Enjoyableness	.464	.597	.374	.739	.39
Difficulty	.556	.716	.566	.814	-.02
Self-efficacy	.595	.748	.615	.835	.01
Usefulness	.631	.776	.657	.853	.02
Relevance	.790	.880	.817	.921	-.05
Total	.779	.873	.805	.917	.07

*r* refers to Pearson’s correlation between SSAS constructs at Time 1 and Time 2

### Sensitivity

Scale sensitivity of the translated SSAS was explored by computing (i) the distribution of responses according to the spectrum of responses categories for each item, (ii) mean, standard deviation, observed range, (iii) item variance and (iv) skewness and kurtosis. Response distribution and mean of each item should confirm no evidence of ceiling effect, namely, response distribution should be predominantly close to the center of the range of possible scores or responses [53]. In addition, items with high variance are desirable, which would confirm that the translated SSAS is capable of discriminate among individuals with different attitudinal profiles towards school science [53]. In relation to skewness and kurtosis, several recommendation from the literature were followed: Kline [82] suggested indices not higher than 3 for skewness and 10 for kurtosis and others have established ±2 as acceptable limits [74,83,84].

The distribution of responses covered all the spectrum of response categories (Table 8). Most of responses were concentrated in the middle range of the scale, however, responses to item 3 (measuring perceived difficulty of school science) were concentrated in the first two responses categories (60.3%), indicating that the majority of students do no perceive school science as a difficulty subject. Similarly, items 6 and 7, measuring usefulness and relevance of school science, respectively, reported responses concentrated in the last two responses categories (78.7% and 73.1%), indicating that in general students perceive school science as useful and relevant.

**Table 8:** Distribution of responses.

*SSAS* *items*	*Response category (%)*				
	*1*	*2*	*3*	*4*	*5*
Item 1	13	8.7	46.1	20.8	11.4
Item 2	8.2	8.2	26.5	34.5	22.6
Item 3	33.1	27.2	21.9	11.2	6.6
Item 4	5.5	8.0	31.7	37.9	16.9
Item 5	7.1	7.8	19.6	26.7	38.8
Item 6	3.4	2.3	15.5	32.4	46.3
Item 7	2.3	4.6	20.1	31.3	41.8
Item 8	6.8	10.7	36.3	29.5	16.7
Item 9	3.0	8.0	32.0	29.0	28.1
Item 10	7.5	11.9	19.6	28.3	32.6

The mean scores of most items were the middle point of the scale, ranging from 2.31 to 4.16 on a scale from 1 to 5 (Table 9). As shown by the negative skewness of all but item 3, responses were weighted towards the positive end of the 5-point response format. However, the values of skewness and kurtosis fall within the acceptable range of ±2 limits [74,83,84], suggesting that the scale did not report evidence of ceiling effects. Based on these results, the Spanish School Science Attitude survey (S-SSAS) has satisfactory sensitivity.

**Table 9:** Descriptive statistics of translated SSAS items.

*SSAS* *items*	*Range*	*M*	*SD*	*Variance*	*Skewness*	*Kurtosis*
Item 1	1–5	3.09	1.12	1.267	-.244	-.319
Item 2	1–5	3.55	1.17	1.360	-.631	-.281
Item 3	1–5	2.31	1.23	1.501	.628	-.582
Item 4	1–5	3.53	1.04	1.078	-.572	.046
Item 5	1–5	3.82	1.23	1.504	-.842	-.240
Item 6	1–5	4.16	1	.995	-1.298	1.519
Item 7	1–5	4.06	1	1.006	-.935	.387
Item 8	1–5	3.38	1.1	1.198	-.353	-.336
Item 9	1–5	3.71	1.1	1.107	-.434	-.409
Item 10	1–5	3.67	1.3	1.568	-.642	-.624

## Conclusion

The purpose of this study was to translate the single-item SSAS survey [21]for its use by elementary Spanish-speaking students and to test its validity and reliability. Through a multistage translation approach and multiple psychometric evaluations, the current study provides evidence that the S-SSAS is a valid and reliable instrument for measuring elementary students’ attitudes toward school science.

Regarding translation validity, the Think-Aloud structured interviews indicated that the S-SSAS is easily understood and interpreted by Spanish students in 3^rd^ to 6^th^ grades of Elementary Education. Additionally, the panel of experts shared acceptable agreement in the assignments of items to each attitudinal construct. These results provide face and content validity for the scale.

Psychometric evaluation of the S-SSAS indicated an adequate level of internal consistency reliability and that response were well distributed along the response categories, showing great sensitivity and no evidence of extreme response tendency. Intraclass correlation coefficient supported the temporal stability reliability of the translated SSAS, with a 10-days span between first and second administration. Pearson correlation coefficient reported acceptable predictive validity with strong correlation between expected attitudinal constructs based on results reported in specialized literature of attitudes, and also great concurrent validity, thus obtaining high correlation between the attitudinal constructs of the S-SSAS scale and two attitudes measures of conceptual convergence already validated in the literature. In addition, discriminative validity between S-SSAS constructs was confirmed and parametric and non-parametric tests indicated that the translated version of the SSAS replicates similar results to those of its original version in terms of sex and rural or urban school variables, thus confirming discriminative validity and further supporting the applicability of the S-SSAS.

One main limitation should be acknowledged. The concurrent validity was assessed using two other scales that weren’t previously validated for Spanish-speaking students. Ideally, S-SSAS results should have been correlated with other Spanish scales in order to obtain more reliable results. However, given the absence of validated Spanish scales for elementary education, we were forced to use two scales that were validated in another language. To counter this limitation, both Abd-El-Khalick et al. [24] and Sabah et al. [60] scales were translated into Spanish using the same multistage translation approach as for the S-SSAS. Future studies should consider subscales from the Spanish version of the TOSRA [19] to further examine the concurrent validity of the S-SSAS. Although the Navarro et al.’s [19] TOSRA was validated with high school students, it’s psychometric properties for Spanish speaking students are more likely to be stronger than those of the instruments used in this study. In addition, future studies should administer the original and the Spanish SSAS to a bilingual sample and compute equivalence tests between both scores, thus further examining the S-SSAS applicability.

Taken together, in can be concluded that the Spanish SSAS represents a first effort to provide a valid and reliable measure to examine attitudes toward school science of Spanish-speaking elementary students, especially when there are time constraints for data collection. This instrument is easy to administer and quick to answer because it consists of only ten single-item measures equally interpretable by both girls and boys from rural and urban schools. Therefore, it can be useful in quasi-experimental time-series designs where several data measurement is needed.

## Supporting information

S1 Table Similarities between SSAS and two external measures used for examining concurrent validity. (PDF)

S2 Table Comparison between researchers and students interpretation of the S-SSAS items. (PDF)

S1 File Administration instructions. (PDF)

## References

[1] Sadler PM, Gerhard S, Zahra H, Tai R. Stability and Volatility of STEM career interest in high school: A gender Study. Sci Educ. 2012;96: 411–427. 10.1002/sce.21007

[2] Smith E. Women into science and engineering? Gendered participation in higher education STEM subjects. Br Educ Res J. 2011;67: 993–1014. 10.1080/01411926 2010.515019

[3] MECD. Datos y cifras del Sistema Universitario español. Curso 2015/2016. Secretaría General Técnica; 2016.

[4] Archer L, Dewitt J, Osborne J, Dillon J, Willis B, Wong B. “Doing” science versus “being” a scientist: Examining 10/11-year-old schoolchildren’s constructions of science through the lens of identity. Sci Educ. 2010;94: 617–639. 10.1002/sce.20399

[5] Kennedy J, Lyons T, Quinn F. The continuing decline of science and mathematics enrolments in Australian high schools. Teach Sci. 2014;60: 34–46.

[6] Osborne J, Simon S, Collins S. Attitudes towards science: A review of the literature and its implications. Int J Sci Educ. 2003;25: 1049–1079. 10.1080/0950069032000032199

[7] Potvin P, Hasni A. Interest, motivation and attitude towards science and technology at K-12 levels: A systematic review of 12 years of educational research. Stud Sci Educ. 2014;50: 85–129. 10.1080/03057267.2014.881626

[8] Blalock CL, Lichtenstein MJ, Owen S, Pruski L, Marshall C, Toepperwein MA. In pursuit of validity: A comprehensive review of science attitude instruments 1935–2005. Int J Sci Educ. 2008;30: 961–977. 10.1080/09500690701344578

[9] Germann PJ. Development of the attitude toward science in school assessment and its use to investigate the relationship between science achievement and attitude toward science in school. J Res Sci Teach. 1988;25: 689–703.

[10] Noll VH. Measuring the scientific attitude. J Abnorm Soc Psychol. 1935;30: 145–154.

[11] Fraser BJ. Development of a test of science-related attitudes. Sci Educ. 1978;62: 509–515. 10.1002/sce.3730620411

[12] Summers R, Abd-El-Khalick F. Development and validation of an instrument to assess student attitudes toward science across grades 5 through 10. J Res Sci Teach. 2018;55: 172–205. 10.1002/tea.21416

[13] Zhang D, Campbell T. The psychometric evaluation of a three-dimension elementary science attitude survey. J Sci Teacher Educ. 2011;22: 595–612. 10.1007/s10972-010-9202-3

[14] Guzey SS, Harwell M, Moore T. Development of an instrument to assess attitudes toward Science, Technology, Engineering, and Mathematics (STEM). Sch Sci Math. 2014;114: 271–279. 10.1111/ssm.12077

[15] Wang TL, Berlin D. Construction and validation of an instrument to measure taiwanese elementary students’ attitudes toward their Science class. Int J Sci Educ. 2010;32: 2413–2428. 10.1080/09500690903431561

[16] Campbell DT, Stanley JC, Cage NL. Experimental and quasi-experimental designs for research. Boston, MA: Houghton, Mufflin and Company; 1963.

[17] De Pro A, Pérez A. Actitudes de los alumnos de Primaria y Secundaria ante la visión dicotómica de la Ciencia. Enseñanza las Ciencias. 2014;32: 111–132. 10.5565/rev/ensciencias.1015

[18] Vázquez Á, Manassero MA. El declive de las actitudes hacia la ciencia de los estudiantes: Un indicador inquietante para la educación científica. Rev Eureka sobre Enseñanza y Divulg las Ciencias. 2008;5: 274–292.

[19] Navarro M, Förster C, González C, González-Pose P. Attitudes toward science: Measurement and psychometric properties of the Test of Science-Related Attitudes for its use in Spanish-speaking classrooms. Int J Sci Educ. Taylor & Francis; 2016;38: 1459–1482. 10.1080/09500693.2016.1195521

[20] Fraser BJ. Test of science-related attitudes. Melbourne: Australian Council for Educational Research; 1981.

[21] Kennedy J, Quinn F, Taylor N. The school science attitude survey: A new instrument for measuring attitudes towards school science. Int J Res Method Educ. Taylor & Francis; 2016;39: 422–445. 10.1080/1743727X.2016.1160046

[22] Klopfer LE. Evaluation of learning in science In: Bloom BS, Hastings JT, Madaus GF, editors. Handbook of formative and summative evaluation of student learning. London: McGraw-Hill; 1971.

[23] Gardner PL. Attitudes to Science: A review. Stud Sci Educ. 1975;2: 1–41.

[24] Abd-El-Khalick F, Summers R, Said Z, Wang S, Culbertson M. Development and large-scale validation of an instrument to assess arabic-speaking students’ attitudes toward science. Int J Sci Educ. 2015;37: 2637–2663. 10.1080/09500693.2015.1098789

[25] Hillman SJ, Zeeman SI, Tilburg CE, List HE. My Attitudes Toward Science (MATS): The development of a multidimensional instrument measuring students’ science attitudes. Learn Environ Res. Springer Netherlands; 2016;19: 203–219. 10.1007/s10984-016-9205-x

[26] Ajzen I. The theory of planned behavior. Organ Behav Hum Decis Process. 1991;50: 179–211.

[27] Wigfield A, Eccles JS. The development of achievement task values: a theoretical analysis. Dev Rev. 1992;12: 265–310.

[28] Wigfield A, Eccles JS. Expectancy-value theory of motivation. Contemp Educ Psychol. 2000;25: 68–81. 10.1006/ceps.1999.1015 10620382

[29] Wigfield A, Eccles JS. The development of competence beliefs, expectancies for success, and achievement values from childhood through adolescence In: Wigfield A, Eccles JS, editors. Development of achievement motivation. San Diego: Academic Press; 2002 pp. 91–120.

[30] Eccles JS, Wigfield A. In the mind of the achiever: the structure of adolescents’ academic achievement related-beliefs and self-perceptions. Personal Soc Psychol Bull. 1995;21: 215–225.

[31] Eccles JS, Wigfield A, Harold RB, Blumenfeld PB. Age and gender differences in children’s self- and task perceptions during elementary school. Child Dev. 1993;64: 830–847. 8339698

[32] Said Z, Summers R, Abd-El-Khalick F, Wang S. Attitudes toward science among grades 3 through 12 Arab students in Qatar: Findings from a cross-sectional national study. Int J Sci Educ. 2016;38: 621–643. 10.1080/09500693.2016.1156184

[33] Munby H. Issues of validity in science attitude measurement. J Res Sci Teach. 1997;34: 337–341.

[34] Munby H. Thirty studies involving ‘Scientific Attitude Inventory’: What confidence can we have in this Instrument? J Res SciTeach. 1983;20: 141–162.

[35] Pérez Manzano A, De Pro Bueno A. Algunos datos sobre la visión de los niños y de las niñas sobre las ciencias y del trabajo científico. iQualRev Género e Igual. 2018;0: 18 10.6018/iQual.306091

[36] Marbá-Tallada A, Márquez Bargalló C. ¿Qué opinan los estudiantes de las clases de ciencias? Un estudio transversal de sexto de Primaria a cuarto de ESO. Enseñanza las ciencias. 2010;28: 19–30.

[37] Pérez-Franco D, de Pro-bueno AJ, Pérez-Manzano A. Actitudes ambientales al final de la ESO. Un estudio diagnóstico con alumnos de Secundaria de la región de Murcia. Rev Eureka sobre Enseñanza y Divulg las Ciencias. 2018;15: 3501 10.25267/Rev

[38] Vázquez Alonso Á, Manassero–Mas MA. El declive de las actitudes hacia la ciencia de los estudiantes: un indicador inquietante para la educación científica. Rev Eureka sobre Enseñanza y Divulg las Ciencias. 2008;5: 274–292.

[39] Vázquez-Alonso Á, Manassero-Mas MA. La relevancia de la educación científica: actitudes y valores de los estudiantes relacionados con la Ciencia y la Tecnología. Enseñanza las Ciencias. 2009;27: 33–48.

[40] Vázquez-Alonso Á, Manassero–Mas MA. Perfiles actitudinales de la elección de ciencias en secundaria según el sexo y el tipo de educación. Rev Electrónica Enseñanza las Ciencias. 2010;9: 242–260.

[41] Vázquez-Alonso Á, Manassero–Mas MA. Imagen de la ciencia y la tecnología al final de la educación obligatoria. Cult y Educ. 2004;16: 385–398.

[42] Vázquez-Alonso Á, Manassero–Mas MA. El descenso de las actitudes hacia la ciencia de chicos y chicas en la educación obligatoria. CiênciaEduc. 2011;17: 249–268.

[43] Vázquez-Alonso Á, Acevedo Díaz JA, Manassero–Mas MA, Acevedo Romero P. Actitudes del alumnado sobre ciencia, tecnología y sociedad, evaluadas con un modelo de respuesta múltiple. RevElectronInvestigEduc. 2006;8: 1–37.

[44] Vázquez-Alonso Á, Manassero–Mas MA. Una evaluación de las actitudes relacionadas con la ciencia. Enseñanza las Ciencias. 1997;15: 199–213.

[45] Schreiner C, Sjøberg S. ROSE: The Relevance of Science Education. Sowing the seeds of Rose. Background, rationale, questionnaire development and data collection for ROSE (The Relevance of Science Education)—a comparative study of students’ views of science and science education. Oslo: Acta Didactica; 2004.

[46] Costello AB, Osborne JW. Best Practices in exploratory factor analysis: Four recommendations for getting the most from your analysis. Pract Assessment, Res Educ. 2005;10: 1–9. doi: 10.1.1.110.9154

[47] Gaskin CJ, Happell B. International Journal of Nursing Studies On exploratory factor analysis A review of recent evidence, an assessment of current practice, and recommendations for future use. Int J Nurs Stud. 2014;51: 511–521. 10.1016/j.ijnurstu.2013.10.005 24183474

[48] Bowling A. Just one question: If one question works, why ask several? J Epidemiol Community Health. 2005;59: 342–345. 10.1136/jech.2004.021204 15831678 PMC1733095

[49] Robins RW, Hendin HM, Trezniewski KH. Measuring global self-esteem: Construct validation of a single-item measure and the Rosenberg Self-Esteem Scale. Personal Soc Psychol Bull. 2001;27: 151–161.

[50] Woods SA, Hampson SE. Measuring the big five with single items using a bipolar response scale. Eur J Pers. 2005;19: 373–390.

[51] Arias MRM, Lloreda MJH, Lloreda MVH. Psicometría. Alianza Editorial; 2014.

[52] Clark LA, Watson D. Constructing validity: basic issues in objective scale development. Psychol Assess. 1995;7: 309–319. 10.1037/1040-3590.7.3.309

[53] DeVellis RF. Scale development Theory and applications. Los Angeles: SAGE; 2017.

[54] Nunnally JC, Bernstein IH. Psychometric theory. New York: McGraw-Hill; 1994.

[55] Mokkink LB, Terwee CB, Patrick DL, Alonso J, Stratford PW, Knol DL, et al The COSMIN study reached international consensus on taxonomy, terminology, and definitions of measurement properties for health-related patient-reported outcomes. J Clin Epidemiol. Elsevier Inc; 2010;63: 737–745. 10.1016/j.jclinepi.2010.02.006 20494804

[56] Polit DF, Yang F. Measurement and the measurement of change: A primer for health professionals. Philadelphia: Lippincott Williams & Wilkins; 2016.

[57] Polit DF. Assessing measurement in health: Beyond reliability and validity. Int J Nurs Stud. Elsevier Ltd; 2015;52: 1746–1753. 10.1016/j.ijnurstu.2015.07.002 26234936

[58] Borsa JC, Damásio BF, Bandeira DR. Cross-cultural adaptation and validation of psychological instruments: some considerations. Paidéia. 2012;22: 423–432.

[59] Erdinç O, Lewis JR. Psychometric evaluation of the T-CSUQ: The Turkish version of the computer system usability questionnaire. Int J Hum Comput Interact. 2013;29: 319–326. 10.1080/10447318.2012.711702

[60] Sabah S, Hammouri H, Akour M. Validation of a scale of attitudes toward science across countries using rasch model: Findings from TIMSS. J Balt Sci Educ. 2013;12: 692–703.

[61] de Vet HCW, Terwee CB, Knol DL, Bouter LM. When to use agreement versus reliability measures. J Clin Epidemiol. 2006;59: 1033–1039. 10.1016/j.jclinepi.2005.10.015 16980142

[62] Shrout PE, Fleis JL. Intraclass Correlations: Uses in assessing rater reliability. Psychol Bull. 1979;86: 420–428. 18839484 10.1037//0033-2909.86.2.420

[63] Koo TK, Li MY. A guideline of selecting and reporting intraclass correlation coefficients for reliability research. J Chiropr Med. Elsevier B.V.; 2016;15: 155–163. 10.1016/j.jcm.2016.02.012 27330520 PMC4913118

[64] Beatty PC, Willis GB. Research synthesis: The practice of cognitive interviewing. Public Opin Q. 2007;71: 287–311. 10.1093/poq/nfm006

[65] Demirel M, Dağyar M. Effects of problem-based learning on attitude: A meta-analysis study. Eurasia J Math Sci Technol Educ. 2016;12: 2115–2137. 10.12973/eurasia.2016.1293a

[66] Palmer TA, Burke PF, Aubusson P. Why school students choose and reject science: A study of the factors that students consider when selecting subjects. Int J Sci Educ. Taylor & Francis; 2017;39: 645–662. 10.1080/09500693.2017.1299949

[67] Schroeder CM, Scott TP, Tolson H, Huang T-Y, Lee Y-H. A meta-analysis of national research: Effects of teaching strategies on student achievement in science in the United States. J Res Sci Teach. 2007;44: 1436–1460. 10.1002/tea.20212

[68] Ceci SJ, Williams WM. Why aren’t more women in science. Top researchers debate the evidence. Washington, DC: American Psychological Association; 2007.

[69] Chachashvili-Bolotin S, Milner-Bolotin M, Lissitsa S. Examination of factors predicting secondary students’ interest in tertiary STEM education. Int J Sci Educ. Taylor & Francis; 2016;38: 366–390. 10.1080/09500693.2016.1143137

[70] Betz NE. Career self-efficacy: Exemplary recent research and emerging directions. J Career Assess. 2007;15: 403–422. 10.1177/1069072707305759

[71] Andersen HM, Krogh LB, Lykkegaard E. Identity matching to scientists: Differences that make a difference? Res Sci Educ. 2014;44: 439–460. 10.1007/s11165-013-9391-9

[72] Sellami A, El-Kassem RC, Al-Qassass HB, Al-Rakeb NA. A path analysis of student interest in STEM, with specific reference to Qatari students. Eurasia J Math Sci Technol Educ. 2017;13: 6045–6067. 10.12973/eurasia.2017.00999a

[73] Field A. Discovering statistics using SPSS. London: SAGE; 2009.

[74] Brown TA. Confirmatory factor analysis for applied research. New York: Guilford Press; 2006.

[75] IBM C. IBM SPSS statistics for Windows. Version 24.0. Armonk, NY: IBM Corp; 2016.

[76] Cortina JM. What is coefficient alpha? An examination of theory and applications. J Appl Psychol. 1993;78: 98–104.

[77] Tavakol M, Dennick R. Making sense of Cronbach’s alpha. Int J Med Educ. 2011;2: 53–55. 10.5116/ijme.4dfb.8dfd 28029643 PMC4205511

[78] Nunnally JC. Psychometric theory. New York: McGraw-Hill; 1978.

[79] Keszei AP, Novak M, Streiner DL. Introduction to health measurement scales. J Psychosom Res. Elsevier Inc.; 2010;68: 319–323. 10.1016/j.jpsychores.2010.01.006 20307697

[80] Terwee CB, Schellingerhout JM, Verhagen AP, Koes BW, De Vet HCW.Methodological quality of studies on the measurement properties of neck pain and disability questionnaires: A systematic review. J Manipulative PhysiolTher. 2011;34: 261–272. 10.1016/j.jmpt.2011.04.003 21621728

[81] Toma RB, Greca IM. The effect of integrative STEM instruction on elementary students’ attitudes toward science. EURASIA J Math Sci Technol Educ. 2018;14: 1383–1395. 10.29333/ejmste/83676

[82] Kline P. The handbook of psychological testing. New York: Routledge; 2000.

[83] Gravetter F, Wallnau L. Essentials of statistics for the behavioral sciences. Belmont, CA: Wadsworth; 2014.

[84] Trochim WM, Donnelly JP. The research methods knowledge base. Cincinnati: Atomic Dog Publishing Inc; 2006.

