# Supplementary material for: Validation of the single-items Spanish-School Science Attitude Survey (S-SSAS) for elementary education
Source: PLoS One. 2019 Jan 2;14(1):e0209027. doi: 10.1371/journal.pone.0209027 (PMC6314597; doi:10.1371/journal.pone.0209027)
Supplement: S1 Table — (PDF) [file pone.0209027.s001.pdf]

S1 Table. Similarities between SSAS and two external measures used for examining concurrent validity

<table>
<tr><th>SSAS [21]</th><th>Scale of Attitude toward Science [60]</th><th>ASSASS [24]</th></tr>
<tr><td>Intention to enroll in further science
1. I am very likely to enroll on a science course in Year 11 (LT)</td><td>-</td><td>Intention to enroll
I would enjoy working in a science-related career
I will continue studying science after I leave school
I will take additional science courses in the future
I will study science if I get into a university
My family encourages me to have a science-related career
I will become a scientist in the future</td></tr>
<tr><td>Enjoyableness of school science
2. I think science is (SD boring – fun)</td><td>Positive Affect toward Science
I enjoy learning science
I like science</td><td rowspan="5">-</td></tr>
<tr><td>Perceived difficulty of school science
3. I struggle with completing the assignments for science class (LT)</td><td rowspan="2">Self-Confidence in learning Science
I usually do well in science
I learn things quickly in science;</td></tr>
<tr><td>Perception of self-efficacy in school science
4. I think I am very good at science (LT)</td></tr>
<tr><td>Usefulness of science to careers
5. A job as a scientist would be interesting (LT)
6. For my planned career, knowledge of school science will be
(SD worthless – required)</td><td rowspan="2">Students’ Valuing Science
I think learning science will help me in my daily life
I need science to learn other school subjects
I would like to do well
to get the job I want</td></tr>
<tr><td>Relevance of school science
7. Science helps to make life better (LT)
8. I want to learn about plants in my area (LT)
9. For my everyday life, I think school science is (SD irrelevant – relevant)
10. I want to learn about electricity and how it is used in the home</td></tr>
</table>

LT: Likert-type items

SD: Semantic differential items

Abd-el-Khalick et al. [24] and Sabah et al. [60] instruments use LT items
