# Supplementary material for: Validation of the single-items Spanish-School Science Attitude Survey (S-SSAS) for elementary education
Source: PLoS One. 2019 Jan 2;14(1):e0209027. doi: 10.1371/journal.pone.0209027 (PMC6314597; doi:10.1371/journal.pone.0209027)
Supplement: S2 Table — (PDF) [file pone.0209027.s002.pdf]

S2 Table. Comparison between researchers and student interpretation of the S-SSAS items

SSAS original items	*Researchers interpretation*	*Students interpretation*
I am very likely to enroll on a science course in Year 11(LT).	This item measures the extent to which the student intends to enroll in higher education science courses.	I think this item asks me if I am going to sign up [enroll] for science in ESO.
I think science is (SD boring – fun).	This item measures the extent to which students find science classes enjoyable.	Here they tell me if I have fun in science classes and if I like it.
I struggle with completing the assignments for science class (LT).	This item measures whether students have difficulty when completing science homework.	In this item I have to say whether or not I find it difficult to do science homework.
I think I am very good at science (LT).	This item measures whether students consider themselves as capable and successful in science.	I believe he asks me if I am good at science. Well, I think I am, because I get good grades in exams.
A job as a scientist would be interesting (LT).	This item measures the extent to which students find the work/job of scientists interesting.	This question refers to whether I find what scientists do [at their job] interesting or not.
For my planned career, knowledge of school science will be (SD worthless – required).	This item examines whether students consider that knowledge learned in science classes can be of value to them for pursuing the career or studies they would like to pursue.	I think this question refers to whether I think I need what I learn in science to study what I want.
Science helps to make life better (LT).	This item measures the extent to which science has a positive impact on people's lives.	It asks me if I believe that science is good for our lives.
I want to learn about plants in my area (LT).	This item measures the extent to which students show interest in learning about plants in their area (city and surroundings).	This question tells me if I want to learn about plants. The plants I see on the street, in my neighborhood...
For my everyday life, I think school science is (SD irrelevant – relevant).	This item measures whether students consider science to be important to their daily lives.	I think it refers to whether in my day to day science is important to me.
I want to learn about electricity and how it is used in the home.	This item measures the extent to which students show interest in learning about electricity and its use at home.	This sentence is about if I want to study electricity and how it works in a house.

* The items used in the interview were the one corresponding to the Spanish translation of the S-SSAS, reported in table 1.
