# Supplementary material for: Validation of the single-items Spanish-School Science Attitude Survey (S-SSAS) for elementary education
Source: PLoS One. 2019 Jan 2;14(1):e0209027. doi: 10.1371/journal.pone.0209027 (PMC6314597; doi:10.1371/journal.pone.0209027)
Supplement: S1 File — (PDF) [file pone.0209027.s003.pdf]

S3 File. Administration instructions

ENGLISH

To ensure validity and reliability of the S-SSAS, please read all administration instructions carefully. By using the S-SSAS scale you are acknowledging that you have read and understand the administration guidelines provided, and that if you have any specific question, you have contacted the authors for further guidelines and assistance. The authors of the S-SSAS assume no responsibility for any use of this instrument other than that described in this study.

Description of the S-SSAS

The S-SSAS is a scale that consists of 10 statements measuring attitudes toward six constructs or aspects related to the school science subject. The examinee is asked to read each statement and to select the answer option that best reflects his/her point of view, without time limit. Each statement was adapted to ensure comprehension of students enrolled in 3rd to 6th grade of Spanish-elementary grades (8 to 12 years old students).

Administration of the S-SSAS

Use the sheet provided at the end of this administration manual. Print this sheet in A-4 paper. If background information is to be included by the examinee (for example, age, name, course), provide an additional page with all the extra questions.

In order to reduce biased response from the examinees, the S-SSAS should not be administered by the classroom teacher. Ideally, an external individual should administer the S-SSAS without the presence of the classroom teacher. If this is not possible, ask a colleague from another class to administer the questionnaire without you being present in the classroom.

Please follow these instructions:

1. Make sure you are familiar with the items included and the response format.
2. Administer the S-SSAS in a quiet room or classroom, free from distractions and interruptions.
3. Introduce the S-SSAS to the examinees by saying:
   This is an opinion questionnaire about the School Science subject. There is no right or wrong answers, only your opinion matters. Your participation is voluntary and your answers will not affect your academic grades. If you do not wish to respond, it is preferable that you let the examiner know it instead of answering randomly. There is no time limit. Once you have finished, double check that you answered every question and leave the questionnaire over your table upside down.
4. Project or write the following example on the board to make sure students know how to answer questions. Explain that a person who likes playing basketball would select the squares that are closer to the "Totally agree" response option, and that a person who does not like playing basketball would select the squares that are closer to the "Totally disagree" option. Make sure no one has any doubts or questions.

**I really like to play basketball.**						
Totally disagree	☐	☐	☐	☐	☐	Totally agree

5. Distribute the instrument to each examinee face down on the table, asking students to wait until your instruction to start.
6. The S-SSAS has been adapted and can be used with lower grade elementary students. However, there may be some students who do not understand some words. Once students have begun to answer the questions, you can only answer question related to words that they cannot understand.

Scoring procedure of the S-SSAS

Add a numeric value to each square from 1 to 5 (from left to right):

Totally disagree	☐	☐	☐	☐	☒	Totally agree
Totally disagree	*1*	*2*	*3*	*4*	*5*	*Totally agree*

A score of 5 represents very favourable and positive attitude. A score of 1 represents very unfavourable and negative attitudes. This is different for item 3, where responses closer to 1 are desired. The first four constructs are measured by only one item per construct. The last three constructs are measured by multiple items per construct. To obtain a score for each construct, sum the items of each construct and divide them by the total number of items within that construct.

- *Intention to enrol in further science (I)*

I = item 1

- *Enjoyableness of school science (E)*

E = item 2

- *Perceived difficulty of school science (D)*

D = item 3

- *Perception of self-efficacy in school science (S)*

S = item 4

- *Usefulness of science to careers (U)*

$$\text{U} = \frac{\text{item 5+item 6}}{2}$$

- *Relevance of school science (R)*

$$\text{Personal relevance} = \frac{\text{item 8+item 9+item 10}}{3}$$

$$\text{(R)} = \frac{\text{item 7 +personal relevance}}{2}$$

ESPAÑOL

Para garantizar la validez y fiabilidad del S-SSAS, lea atentamente todas las instrucciones de administración. Al utilizar la escala S-SSAS, usted reconoce que ha leído y comprendido las directrices de administración proporcionadas y que si tiene alguna pregunta específica, se ha puesto en contacto con los autores para obtener asistencia. Los autores del instrumento S-SSAS no asumen ninguna responsabilidad por cualquier uso de este instrumento que no sea el descrito en este estudio.

Descripción del S-SSAS

El S-SSAS es una escala que consta de 10 afirmaciones que miden las actitudes de los estudiantes de Educación Primaria hacia seis constructos o aspectos relacionados con la asignatura Ciencias de la Naturaleza. Se pide al examinando que lea cada afirmación y seleccione la opción de respuesta que mejor refleje su punto de vista, sin límite de tiempo. Cada afirmación fue adaptada para asegurar la comprensión de los estudiantes matriculados desde 3º hasta 6º curso de Educación Primaria (estudiantes de 8 a 12 años).

Administración del S-SSAS

Utilice el modelo de administración proporcionado al final de este manual. Imprima este modelo en papel de tamaño A-4. Si el examinando debe incluir otra información (por ejemplo, nombre, edad, curso, etc.), proporcione una hoja adicional con todos los elementos y cuestiones secundarias.

Para reducir respuestas sesgadas por parte de los examinandos, el S-SSAS no debe ser administrado por el maestro del aula. Idealmente, una persona externa será la encargada de administrar el S-SSAS sin la presencia del profesor del aula. Si esto no es posible, pídale a un colega de otra clase que administre el cuestionario sin que usted esté presente en el aula.

Por favor, siga estas instrucciones:

1. Asegúrese de estar familiarizado con las afirmaciones incluidas y el formato de respuesta.
2. Administre el S-SSAS en un lugar tranquilo, libre de distracciones e interrupciones.
3. Presente el S-SSAS a los examinandos diciendo:
   Se trata de un cuestionario de opinión sobre la asignatura Ciencias de la Naturaleza. No hay respuestas correctas o incorrectas, tan solo importa tu opinión. Tu participación es voluntaria y tus respuestas no afectarán tus notas académicas. Si no deseas responder, es preferible que se lo digas al examinador/profesor en lugar de responder al azar. No hay límite de tiempo. Una vez que hayas terminado, verifica dos veces que hayas contestado a todas las preguntas y deja el cuestionario sobre la mesa bocabajo.
4. Proyecte o escriba el siguiente ejemplo en la pizarra para asegurarse de que los estudiantes sepan cómo responder a las afirmaciones incluidas en el S-SSAS. Explique que una persona a la que le gusta jugar al baloncesto seleccionaría los cuadrados que están más cerca de la opción de respuesta "Totalmente de acuerdo", y que una persona a la que no le gusta jugar al baloncesto seleccionaría los cuadrados que están más cerca de la opción "Totalmente en desacuerdo". Asegúrese de que nadie tenga dudas o preguntas.

**Me gusta mucho jugar al baloncesto.**						
Totalmente en desacuerdo	☐	☐	☐	☐	☐	Totalmente de acuerdo

5. Distribuya el instrumento a cada examinando bocabajo, pidiendo que esperen hasta que usted les dé las instrucciones pertinentes para comenzar.
6. El S-SSAS ha sido adaptado para estudiantes de Educación Primaria. Sin embargo, es posible que algunos estudiantes muestren dificultad para entender algunas palabras. Una vez que los estudiantes hayan comenzado a contestar las afirmaciones, solo responda a preguntas relacionadas con la comprensión de las palabras.

Procedimiento de evaluación del S-SSAS

Añada un valor numérico a cada cuadrado, del 1 al 5 (de izquierda a derecha):

Totalmente en desacuerdo	☐	☐	☐	☐	☒	Totalmente de acuerdo
Totalmente en desacuerdo	*1*	*2*	*3*	*4*	*5*	*Totalmente de acuerdo*

Una puntuación de 5 representa una actitud muy favorable y positiva. Una puntuación de 1 representa una actitud muy desfavorable y negativa. Esto es diferente para la afirmación 3, donde se desean respuestas más cercanas a 1. Los cuatro primeros constructos del S-SSAS se miden empleando una sola afirmación por cada constructo. Los tres últimos constructos del S-SSAS se miden empleando varias afirmaciones por cada constructo. Para obtener una puntuación para cada constructo, sume la(s) afirmación(es) de cada constructo y divida por el número total de afirmaciones existentes en dicho constructo.

- *Intención de matricularse en Ciencias de la Naturaleza en la ESO (I)*

I = afirmación 1

- *Disfrute de Ciencias de la Naturaleza (D)*

D = afirmación 2

- *Dificultad percibida de Ciencias de la Naturaleza (DP)*

DP = afirmación 3

- *Percepción de la auto-eficacia en Ciencias de la Naturaleza (A)*

A = afirmación 4

- *Utilidad de Ciencias de la Naturaleza para las carreras (U)*

$$\mathrm{U} = \frac{\text{afirmación 5} + \text{afirmación 6}}{2}$$

- *Relevancia de Ciencias de la Naturaleza (R)*

$$\text{Relevancia personal} = \frac{\text{afirmación 8} + \text{afirmación 9} + \text{afirmación 10}}{3}$$

$$(\mathrm{R}) = \frac{\text{afirmación 7} + \text{relevancia personal}}{2}$$

A continuación, encontrarás varias frases sobre la asignatura Ciencias de la Naturaleza. Marca con una X el cuadrado que mejor represente tu respuesta.

1. Es muy probable que me apunte a Ciencias de la Naturaleza en la ESO.

Totalmente en desacuerdo	☐	☐	☐	☐	☐	Totalmente de acuerdo

2. Pienso que Ciencias de la Naturaleza es:

Aburrida	☐	☐	☐	☐	☐	Divertida

3. Me cuesta terminar las tareas para la clase de Ciencias de la Naturaleza.

Totalmente en desacuerdo	☐	☐	☐	☐	☐	Totalmente de acuerdo

4. Pienso que soy muy bueno en Ciencias de la Naturaleza.

Totalmente en desacuerdo	☐	☐	☐	☐	☐	Totalmente de acuerdo

5. Un trabajo como científico sería interesante.

Totalmente en desacuerdo	☐	☐	☐	☐	☐	Totalmente de acuerdo

6. Para mis futuros estudios, el conocimiento de las clases de Ciencias de la Naturaleza es:

Inútil	☐	☐	☐	☐	☐	Útil

7. La ciencia ayuda a mejorar la vida.

Totalmente en desacuerdo	☐	☐	☐	☐	☐	Totalmente de acuerdo

8. Quiero aprender sobre las plantas de mi entorno.

Totalmente en desacuerdo	☐	☐	☐	☐	☐	Totalmente de acuerdo

9. Para mi vida diaria, creo que Ciencias de la Naturaleza es:

Poco importante	☐	☐	☐	☐	☐	Muy importante

10. Quiero aprender sobre la electricidad y saber cómo se usa en una casa.

Totalmente en desacuerdo	☐	☐	☐	☐	☐	Totalmente de acuerdo
